# Complex genomic rearrangements: an underestimated cause of rare diseases

**DOI:** 10.1016/j.tig.2022.06.003

**Published:** 2022-07-09

**Authors:** Jakob Schuy, Christopher M. Grochowski, Claudia M.B. Carvalho, Anna Lindstrand

**Affiliations:** 1 Department of Molecular Medicine and Surgery and Center for Molecular Medicine, Karolinska Institutet, Stockholm, Sweden; 2 Department of Molecular and Human Genetics, Baylor College of Medicine, Houston, TX, USA; 3 Pacific Northwest Research Institute, Seattle, WA, USA; 4 Department of Clinical Genetics, Karolinska University Hospital, Stockholm, Sweden

## Abstract

Complex genomic rearrangements (CGRs) are known contributors to disease but are often missed during routine genetic screening. Identifying CGRs requires (i) identifying copy number variants (CNVs) concurrently with inversions, (ii) phasing multiple breakpoint junctions *in cis*, as well as (iii) detecting and resolving structural variants (SVs) within repeats. We demonstrate how combining cytogenetics and new sequencing methodologies is being successfully applied to gain insights into the genomic architecture of CGRs. In addition, we review CGR patterns and molecular features revealed by studying constitutional genomic disorders. These data offer invaluable lessons to individuals interested in investigating CGRs, evaluating their clinical relevance and frequency, as well as assessing their impact(s) on rare genetic diseases.

## CGRs in the human genome

CGRs (see Glossary) are defined as **structural variants (SVs)** that harbor more than one breakpoint junction and/or comprise structures made up of more than one SV *in cis* [1,2]. Larger aberrations known as **complex chromosomal rearrangements (CCRs)** comprise structural rearrangements that have at least three cytogenetically visible breakpoints and represent exchanges of chromosomal sections between more than two chromosomes [3]. For simplicity, the two types of complex genomic structures are discussed together.

CGRs involving large genomic segments (>5 Mb) have been detected by karyotyping of individuals with rare diseases (Box 1) over many years [4]. Such events are rare, exemplified by 0.0026% (*N* = 7) of 269 371 prenatal samples harboring *de novo* presumably balanced CCRs [5]. Historically, CGR breakpoints were inferred using fluorescence *in situ* hybridization (FISH), multi-color banding, as well as karyotyping and **chromosomal microarray (CMA)** [6]. Because **genome sequencing (GS)** methodologies have increased our ability to detect and interpret complex genomic events, a growing number of reports of clinically relevant CGRs with an unexpected level of complexity have been published in the scientific literature [7–12]. Hence, CGRs, that were once presumed to be ‘ultra-rare’ occurrences may be more common than originally thought. This is at least in part due to the increasing ability of third-generation GS technologies to produce longer reads, making it possible to detect rearrangements *in cis* as well as to resolve complex genomic regions [i.e., repeat elements, **segmental duplications (SDs)**, centromeres, and telomeres].

Although no genomic analysis method provides a ‘complete’ view of the genome, each technology has strengths and weaknesses in detecting a given type of SV [13]. Classical chromosome banding analysis under a light microscope (i.e., karyotyping) offers a genome-wide view of aberrations that occur at a resolution of 5–10 Mb [14]. CMAs allow a more comprehensive view of copy number variation at a variable resolution depending on the probe density (1–100 kb genome-wide); however, they cannot detect copy number neutral events known as **balanced chromosomal rearrangements (BCRs)**. Short-read GS detects large and small **copy number variants (CNVs)** depending on the variant callers used and have the potential to detect some BCRs (translocations and inversions). It also allows characterization of breakpoints, often at the nucleotide level. However, short-read GS will not phase complex rearrangement breakpoints nor bridge across genomic regions with poor mappability. Longer DNA molecules are necessary to call and phase such events either through linked, long-read or optical mapping technologies [15], although CNV information is limited using those methodologies. Hence, although each method adds to our understanding of the genomic make-up of a specific sample, there are still gaps in our ability to resolve complex or cryptic genetic aberrations, particularly if only one approach is used.

In fact, recent studies demonstrate that resolving the structure of derivative chromosome(s) or derivative structures from a CGR (or CCR) benefits from applying multiple technologies, for instance molecular cytogenetics methods such as **array comparative genomic hybridization (aCGH)** together with short- and long-read GS. Such an approach has been successfully used in a cohort of patients with specific rare genetic diseases [16,17] and in studies of patients with rare diseases without a molecular diagnosis [18]. Furthermore, using such complementary methodology has revealed additional complexities in seemingly simple chromosome rearrangements [10–12]. In all, using standardized analysis approaches, CGRs are not only underdetected but are also insufficiently characterized, making clinical interpretation challenging. In this review, both obstacles and ‘success stories’ in solving complex rearrangements are presented, as well as insights to guide researchers and clinicians when a CGR is suspected.

## Complex genomic rearrangements in large cohort studies

Large cohort studies have reported common SVs in the general population (Box 2). These cohorts included >1000 individuals and reported unique SVs in total as well as per individual (Table 1). The high number of participants also enabled the detection of a small fraction of CGRs. However, owing to the application of different reference genomes (GRCh37, GRCh38), sequencing depths (7.4× to 105×), and sequencing methodologies including differences in library preparations, the resulting numbers cannot be compared directly. The definition of a rare SV ranges from <1% to below 0.01% minor allele frequency, making refiltering of data with a common filter mandatory before meaningful comparisons and conclusions can be drawn. Moreover, some projects report their SV findings as a total number as well as per genome and per group of SVs (Table 1), whereas others focus on the overall picture [19], the methodology [20], or selected complex cases [21]. Finally, depending on the technology used, some SV types such as inversions may not be detected (false negatives), are detected in excess (false positives) or incorrectly interpreted [22]. Hence, CGRs, many of which involve inversions [23], could be missed. In fact, CGRs are rarely reported and are only available through some of the publications (Table 1).

CGRs are often not in focus and, as a consequence, rarely reported. However, more CGRs are detected when studied using high-resolution methodology, such as in gnomAD where 1.6% (5295/335 470) of the resolved SVs are in fact CGRs [23]. CGRs may be even more abundant because higher CGR fractions were observed by both Collins *et al*. (2.5% of all SVs detected in 689 individuals) [24] and Abel *et al*. (3.3% of rare SVs in 17 795 individuals) [25]. One could argue that these numbers are not comparable because sequencing depth and data analysis were not matched. Nonetheless, similar CGR fractions are also observed in other studies with a comparable approach, such as in Belyeu *et al*. (3.3% of *de novo* SVs in 869 individuals) [26], which suggests that CGRs do represent a small but significant fraction of all SVs.

In summary, the highlights taken from large cohort studies are (i) >20 000 SVs per genome are detected on average with current SV detection technology [22,27], (ii) SVs occur non-randomly in the genome and cluster in repetitive and subtelomeric regions [23,28], (iii) most SVs are deletions and insertions [22,23], and (iv) on average, 2.6% of all SVs are complex [22–24,29,30].

## Clinical challenge: detecting and interpreting complex rearrangements

Most CGRs are detected unexpectedly when an individual comes in for routine testing because of a suspected genetic disorder. Often, most clinical workflows are not able to adequately classify the pathogenicity of these variants, and characterize them simply as ‘complex’ while the underlying mechanism and phenotypic impact remain unknown. Although the number of rare disease cases that appear to be caused by a CGR is increasing, the details pertaining to the full genomic characterization of a given CGR are usually lacking. Failing to properly identify a given SV during genetic testing may lead to incomplete diagnosis and underappreciation of the genetic burden in a patient. For instance, an important aspect of any CGR identified in the clinic is whether it only affects one chromosome (intrachromosomal) or includes several chromosomes (interchromosomal). In general, interchromosomal rearrangements refer to those where multiple non-homologous chromosomes are involved as well as marker chromosomes (i.e., additional chromosomes that result from the fusion of chromosomes). However, some seemingly intrachromosomal CGRs are in fact formed post-zygotically and involve exchange of material between two homologous chromosomes [31]. These rearrangements might harbor loss of heterozygosity segments with the risk of imprinting defects [32,33] or recessive genetic disease [34,35].

When investigating the underlying cause of disease in patients, clinical laboratories need to consider (i) the costs and limitations of each technology concerning false positives and false negatives per type of genetic variant, (ii) the reference genome used (Box 3), (iii) the databases of DNA variants used to filter commons alleles, and (iv) the added value of sequencing parental samples. Technology limitation is the main challenge in properly characterizing the frequency of CGRs in a given cohort, and this is often uncovered when cases are reanalyzed using a new methodology. For example, insertional translocations were reported to occur at 1:10 000 based on karyotyping/FISH studies [36]. Over time, FISH combined with aCGH showed that insertional translocations occur more often, namely 1:500 [37]. In another example, the expected CGR occurrence rates were corrected from 2.8% to 19.2% in karyotypically balanced SVs [38].

Today, because short-read GS is starting to replace exome sequencing in rare disease diagnostics, it is possible to solve many CGRs to nucleotide resolution [8,12]. The process, however, can be labor-intensive because the called variants need to be inspected and assembled manually, and the exact coordinates of the junction must then be confirmed by breakpoint junction PCR. The precise junction information is necessary to phase multiple breakpoints, to position the rearranged genomic segments, and to pinpoint the interrupted gene(s). All of which are crucial for clinical interpretation because the emergence of disease is location-dependent through various mechanisms such as increased or decreased gene copy number [39–41], gene disruptions [12], or perturbed gene regulation [1,42,43]. An average human genome is thought to contain 2.9 rare (<1%) coding SVs on average, and 2% of all people carry rare SVs >1 Mb in size encompassing both balanced and complex rearrangements [25]. Some of these background SVs may play a role in disease pathogenesis, but it is often difficult to properly assess their clinical significance. Moreover, rearrangements that occur in non-coding regions are underappreciated and their effect on regulatory and other potentially pathogenic elements is largely unknown. Databases such as ExAC and gnomAD provide population-wide information about **single nucleotide variants (SNVs)**, enabling statistical calculations of the likelihood that a gene will cause disease, for example, pLoF (probability to cause loss of function), pLI (probability for intolerance to heterozygous pLoF variants), and LOEUF (ratio of pLoF observed/expected) [44]. Such measurements have not yet been fully established for SVs [23]. There have been attempts to calculate similar scores for CNVs [45], but these values cannot be compared to the existing LOEUF because the prediction model for SVs is more complex than for SNVs.

## Genomic disorders provide clues to the molecular features of pathogenic CGRs

The full contribution of pathogenic CGRs to rare diseases is still unknown and we hypothesize that it is underestimated. In a study of *de novo* SVs, an enrichment for *de novo* CGRs was observed (29/869 affected vs 1/61 unaffected) [26]. This hypothesis is also supported by our own work where 3 of 100 consecutive cases referred for CMA in fact harbored CGRs when assessed using GS, revealing that 23% (3/13) of the disease-causing SVs are complex [46]. In addition, Pettersson *et al*. presented evidence that 17% (3/18) of presumably simple large cytogenetically detected inversions harbor additional genomic complexities [10].

Data from **genomic disorders** [42] further support the hypothesis that pathogenic CGRs are highly relevant in genetic diseases caused by SVs, for instance malformation syndromes, intellectual disability, and neurodevelopmental disorders [2]. A growing number of syndromes (https://www.deciphergenomics.org/disorders/syndromes/karyotype) have been identified where recurrent deletions and reciprocal duplications lead to disease through aberrant gene dosage [47], whereas >70 syndromes are caused by nonrecurrent SVs [48]. CGRs are observed in several such syndromes, particularly those with breakpoints mapping to **low-copy repeats (LCRs)** such as Smith–Magenis syndrome [Mendelian Inheritance in Man (MIM) reference #182290], Charcot–Marie–Tooth disease type 1A (MIM #118220), and X-linked ichthyosis (MIM #308100) (reviewed by Carvalho and Lupski [2]).

**Figure 1.**
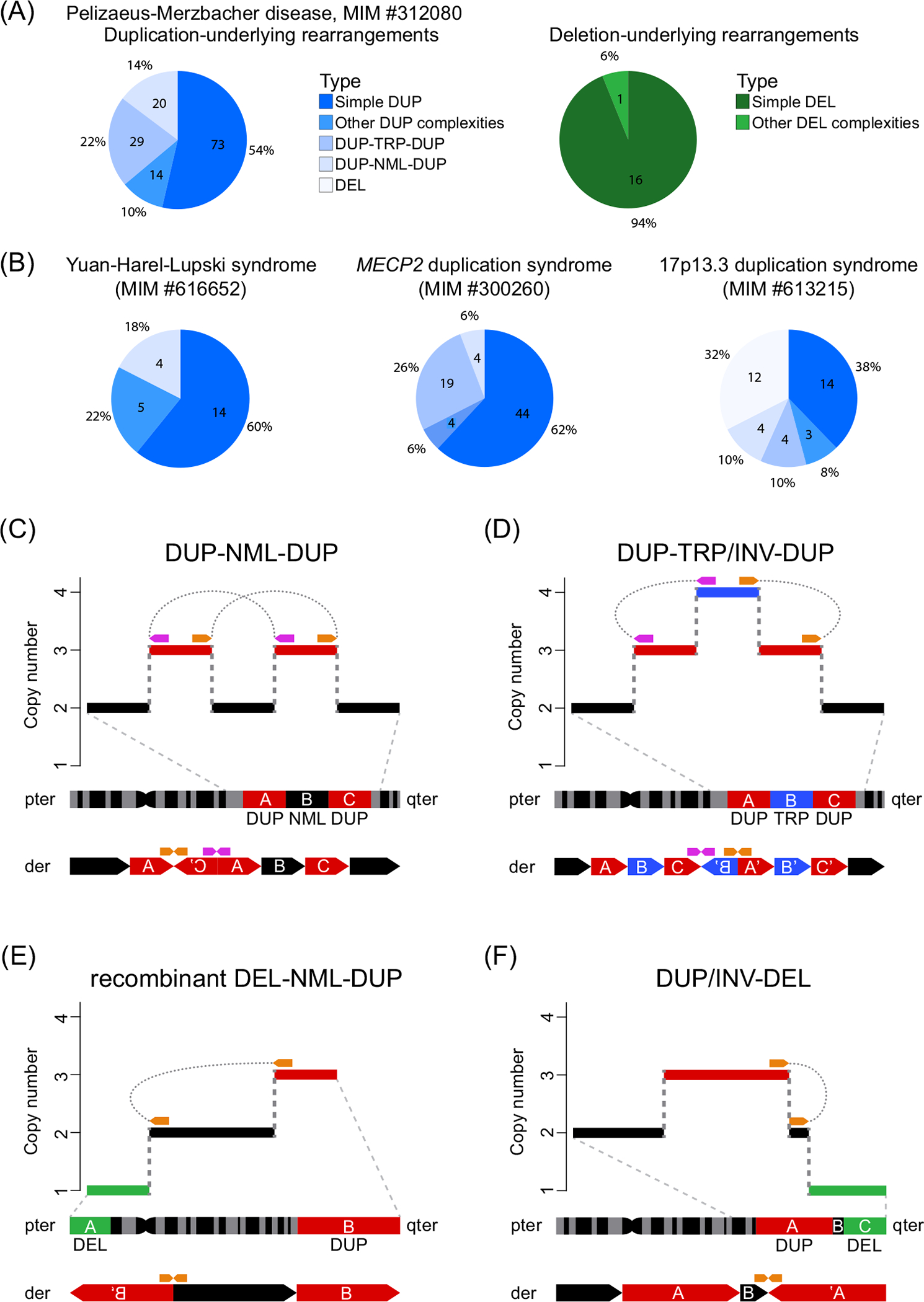
Recurrent patterns of complex genomic rearrangements (CGRs) in constitutional and cancer genomes. (A) Pie charts showing the proportion and absolute numbers of duplications (left) [50,52,92–95] and deletions (right) [96] causing Pelizaeus–Merzbacher disease. (B) Deletions and duplications in complex genomic events causing Yuan–Harel–Lupski syndrome [97], *MECP2* duplication syndrome [41,51,98], and 17p13.3 duplication syndrome [49]. (C) Copy number signature of DUP–NML–DUP (interspersed duplications). One of four predicted DUP–NML–DUP genomic structures [49] is displayed at the bottom; this specific type was experimentally observed in pericentric inversions [10,99]. (D) Copy number signature of the DUP–TRP/INV–DUP CGR [inverted triplication (blue) flanked by duplications (red)]. The derivative structure displayed in the bottom was proposed from aCGH, Sanger sequencing, and FISH experiments. It is the first structure identified in probands affected with *MECP2* duplication syndrome or Pelizaeus–Merzbacher disease [41]. Recently, three alternative structures were proposed based on experimental observations in cancer genomes [55]. (E) Copy number signature of a recombinant DEL–NML–DUP (telomeric deletion followed by a copy number neutral chromosome with a telomeric duplication). The duplicated sequence (red) is inverted and inserted at the location of the deletion (green). This rearrangement results from a meiotic recombination in a parent carrying a heterozygous copy number neutral pericentric INV [10] which will be resolved as a recombinant chromosome with a DEL–NML–DUP structure. (F) Copy number signature of DUP/INV–DEL (inverted duplication adjacent to terminal deletion). This structure results from chromosomes with terminal deletions further repaired by a fold-back mechanism mediated by short segments of homology creating a spacer [B] between the inverted duplications [A]. This type of structure can also be resolved as a translocation (inverted duplication translocation) or as a ring chromosome, both of which can be generated through a breakage–fusion–bridge cycle [100]. Purple and orange arrows represent the location of junctions in the reference genome. Abbreviations: aCGH, array comparative genomic hybridization; der, derivative chromosome; DUP, duplication; FISH, fluorescence-*in*-*situ*-hybridization; INV, inversion; MIM, Mendelian Inheritance in Man; NML, normal; pter, end of the short arm (p) of the chromosome; qter, end of the long arm (q) of the chromosome; TRP, triplication.

A few genomic disorders have been studied extensively to characterize the breakpoints of the pathogenic SVs, including Pelizaeus–Merzbacher disease (PMD, MIM #312080) (Figure 1A), Yuan–Harel–Lupski syndrome (MIM #616652), *MECP2* duplication syndrome (MIM #300260), and 17p13.3 duplication syndrome (MIM #616652) (Figure 1B). These disorders present a high frequency of pathogenic CGRs, the two most common being duplication–normal–duplication (DUP–NML–DUP) (6–18%) and duplication–triplication/inversion–duplication (DUP–TRP/INV–DUP) (10–26%). DUP–TRP/INV–DUP CGRs are often generated by inverted LCR pairs or inverted *Alu* pairs because they can act as recombinant substrates, whereas the DUP–NML–DUP structure can harbor cryptic inversion events [41,49,50], highlighting the remarkable contribution of inversions to the formation of CGRs. Moreover, these studies revealed that application of higher-resolution technologies can uncover additional complexities that were unanticipated in a high fraction of samples (Figure 1) [16,51,52].

From the clinical standpoint, CGRs were observed to contribute to pleiotropy in psychiatric diseases, phenotypic severity in neurodevelopmental disorders, as well as being the cause of common disease – as is the case for DUP–TRP–DUP structures in Parkinson’s disease patients [41,50,53,54]. Both DUP–TRP–DUP and DUP–NML–DUP CGRs are also observed in cancer genomes, highlighting that they can be generated in both somatic and germline cells (Figure 1) [55,56].

## Resolving the genomic architecture of CGRs utilizing multiple methodologies

**Figure 2.**
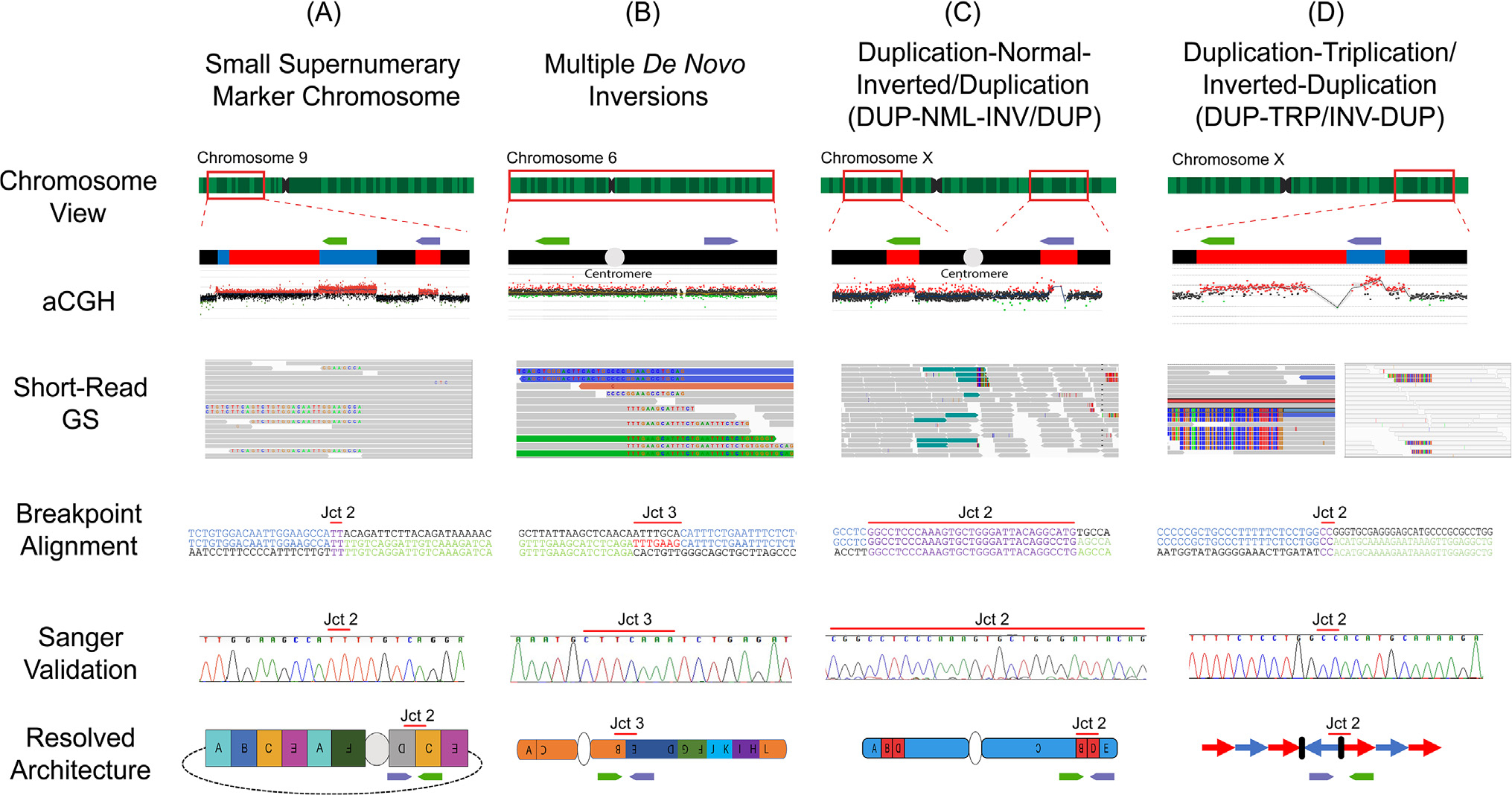
Resolving genomic aberrations through multiple methodologies. Four cases from the literature are presented in which the complete genomic architecture could be resolved through a combination of aCGH, short-read GS, breakpoint sequence alignment, and finally Sanger validation of one of the exemplified breakpoint junctions. These cases include (A) a supernumerary marker chromosome [53], (B) an individual with multiple *de novo* inversions affecting a single chromosome [12], (C) a complex pericentric inversion accompanied by CNVs at the junctions DUP–NML–INV/DUP [10], and (D) a DUP–TRP/INV–DUP at the *MECP2* locus [41]. The relative positions and orientations of primers used to amplify each junction are shown as green and purple arrows in the CNV view. Abbreviations: aCGH, array comparative genomic hybridization; CNV, copy number variation; DUP, duplication; GS, genome sequencing; INV, inversion; Jct, junction; NML, normal; TRP, triplication.

CGRs often involve CNVs and inversions, sometimes translocations and **runs of homozygosity** (ROHs). Therefore, a variety of different methodologies may be warranted to enable the characterization of CGRs and ultimately establish genotype–phenotype correlations. In Figure 2, selected CGR cases are presented that illustrate the use of multiple technologies to characterize the derivative chromosomes down to nucleotide-level resolution, bringing relevant information to clinicians, researchers, as well as families. A brief summary of the four examples is provided below.

- *De novo* supernumerary marker chromosome 9. This marker consisted of segments derived from the short arm of chromosome 9, observed in a family with pleiotropic psychiatric phenotypes in which the marker segregated with disease (Figure 2A). aCGH confirmed multiple copy number gains scattered along the chromosome. The visualization of each position of copy number change in the short-read GS data showed soft-clipped reads, indicative of breakpoint junctions, that were further validated by Sanger sequencing. Finally, droplet digital PCR showed that two junctions occurred twice, which facilitated a complete genomic architectural map of the maker chromosome and gave insights into a possible **chromoanasynthesis**-type mechanism in its formation [53].
- Multiple *de novo* inversions. Multiple inversions occurring on chromosome 6 were first detected through traditional karyotyping (Figure 2B). Because inversions may be copy number neutral events, aCGH provides no information that a genomic aberration is present. Genomic optical mapping data combined with short-read GS provided directionality and orientation of each genomic fragment as well as nucleotide-level resolution of the breakpoint junctions, all of which mapped to the short and long arms of chromosome 6. Combined analysis revealed additional inversions that were not observed by the initial karyotyping, and enabled the discovery of *ARID1B* disruption as the underlying cause of the clinical phenotypic presentation (Coffin–Siris syndrome, MIM #135900) [12].
- Pericentric DUP-NML-INV/DUP. Pathogenic complex recombinant chromosomes may recur multiple times in a family across generations because of the presence of pericentric inversions in the heterozygous state. Combined karyotyping, customized aCGH, and short-read GS revealed pericentric genomic inversions that were generated concomitantly with copy number variation (Figure 2C). The combination of methods used enabled architectural mapping of the aberration with the copy number changes that accompanied the inversion event [10].
- DUP–TRP/INV–DUP. These structures were first described at Xq, spanning *MECP2* (leading to *MECP2* duplication syndrome) and *PLP1* (leading to PMD), mostly affecting males. This genomic structure may also lead to imprinting errors when it occurs adjacent to ROH [17]. This event can be mediated by inverted repeat pairs that act as a recombinant substrate, generating two template switches to form this complex SV. The repetitive features of the genomic loci where such structures are often observed in chromosome Xq required the combined use of approaches such as FISH, Southern blotting, aCGH, and GS. Importantly, probands carrying such CGR presented a more severe phenotype if the triplications spanned the dosage-sensitive genes *MECP2* or *PLP1* [41,50].

These four clinically relevant CGRs demonstrate the importance of using multiple methodologies to resolve the genotypic architecture, and which led to a newly proposed clinical treatment [57], resolved a Mendelian family history, informed genetic counseling, and revealed the underlying cause of variability in disease expression.

**Figure 3.**
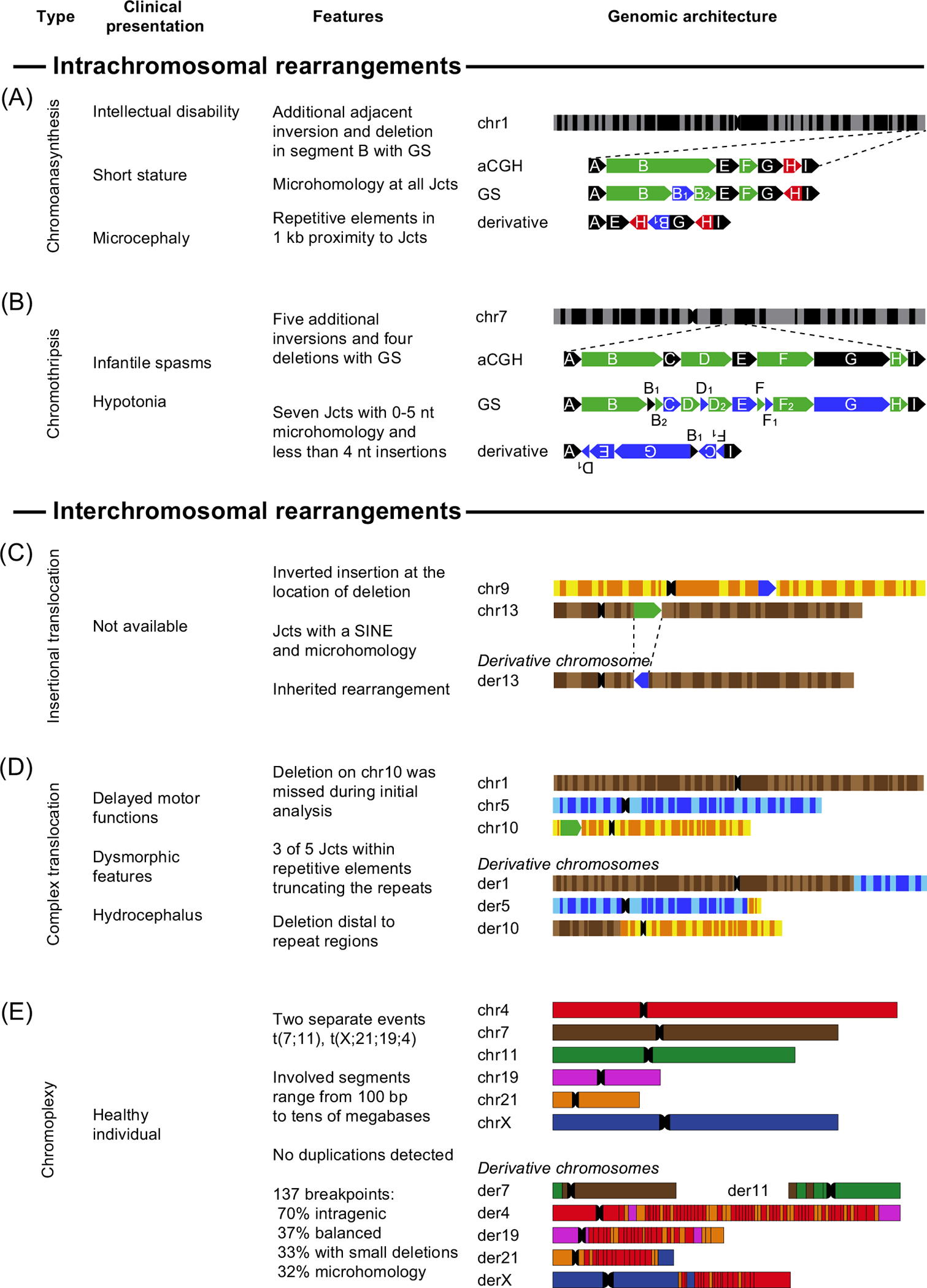
Nonrecurrent patterns of highly complex genomic rearrangements (CGRs) can be classified as intrachromosomal or interchromosomal events. (A) Chromoanasynthesis: an intrachromosomal rearrangement on chromosome 1 first analyzed with aCGH and resolved by GS (patient P2109_162 [7]). The CGR contained multiple deletions (green) and one duplication (red) as well as a hidden inversion (blue) and a deletion which was later revealed by GS. (B) Chromothripsis: an intrachromosomal chromosome 7 rearrangement first analyzed with aCGH. Linked-read GS uncovered five additional inversions and three deletions (patient 00 [7]). (C) Inserted translocation: one individual carried (Cplex9 [101]) an altered chromosome 13 with an inserted segment (blue) from chromosome 9 at the location of the deletion (green). (D) Complex translocation. The fourth case (case 2 in [8]) is a complex translocation between chromosomes 1, 5, and 10, whereas chromosome 10 carries an additional deletion (green). (E) Chromoplexy: the last case [9] is a highly complex rearrangement with 137 breakpoints affecting chromosomes 4, 7, 11,19, 21, and X. Abbreviations: aCGH, array comparative genomic hybridization; der, derivative chromosome; chr, chromosome; GS, genome sequencing; Jct, junction; SINE, short interspersed nuclear element.

Combined approaches are even more relevant to individuals carrying intrachromosomal or interchromosomal CGRs such as chromothripsis, **chromoplexy, chromoanasynthesis,** complex chromosomal insertions, and complex translocations (Figure 3).

## Mechanisms of simple and complex genomic rearrangements

Characterizing an SV at nucleotide-level resolution of the breakpoint junction reveals features of the underlying repair mechanism(s) that lead to a given rearrangement. These mechanisms (and combinations of them) can generate both simple SVs and CGRs.

In many recurrent SVs, repetitive regions of the genome such as LCRs [58,59] and/or SDs [60,61] mediate rearrangements through **nonallelic homologous recombination (NAHR)**. Other repetitive elements, that may act as recombinant substrates and cause genomic rearrangements, include (but are not limited to) sequences from short and long interspersed nuclear elements (SINEs/LINEs), as well as from human endogenous retroviruses (HERVs) [62]. *Alu* elements, a common subclass of SINEs representing 11% of the genome [63], can also generate *Alu*/*Alu*-mediated rearrangements [64]. More complex SVs, often involving alternating copy number variation, occur through errors in replicative repair such as **microhomology-mediated breakinduced replication (MMBIR)** (reviewed by Carvalho and Lupski [2]). The microhomologies at these breakpoints, usually <10 bp, are not predicted to play a role in homologous recombination because their length is less than the minimal efficient processing segment [52,65]. **Non-homologous end joining (NHEJ)** has been proposed to explain the repair of double-strand breaks which occur during catastrophic chromatin disruptions including chromothripsis and chromoplexy, whereas replication-based mechanisms have been proposed to underlie chromoanasynthesis events [66].

## The ‘toolbox’ to resolve CGRs

The major challenge in solving CGRs is to obtain sufficient data without blindly (and costly) applying all available methods. There is no gold standard and, because of the uniqueness of genomic variants, the aim is to tackle the key obstacle to solve the rearrangement – for example, the number of breakpoints in chromothripsis. Therefore, it is advisable to combine at least two technologies that can compensate for the limitations of each other, such as short-read GS/optical mapping or aCGH/long-read GS.

There are key differences between research and clinical diagnostics when analyzing genomic variation. On the one hand, using new methods may be time-consuming and often cost-ineffective, and this approach is mainly used when there is a low number of participants. On the other hand, screening for shared variation using the same accessible tissue as well as taking the phenotype into account leads to a compromise between costs, genomic resolution, and time management.

The choice of method also depends on the purpose of the study. In a screen for commonly shared genomic variation without a pathogenic phenotype, a high vertical **coverage** (sequencing depth) is not as necessary as horizontal coverage (reference coverage). It is suggested to use paired-end GS for a first approach to replace CMA in the clinic [8]. GS also provides single-nucleotide resolution, and available pipelines have been optimized for a good trade-off between high sensitivity and high specificity [67]. Long-read GS and optical mapping are optimal methods for analyzing repetitive regions or longer stretches of cryptic sequences in poorly mapped regions [22]. Moreover, these two methods are suitable for phasing and *de novo* assembly, and thus provide an excellent supplement to short-read GS. In a similar combination approach, data from short- and long-read GS can be merged to form a **hybrid assembly**. This fusion overcomes the disadvantages of both techniques, such as unmappable repetitive sequences in short-read data or low vertical coverage in long-read GS, to build a precise alignment of the individual. This can reduce the error rate to below 10%, as reported in previous studies [68]. Rearrangements with a higher degree of complexity, for example DEL-NML-DUP with overlapping deletion and duplication (that can be visualized through manual inspection of the data), should be confirmed by a second technique such as long-read or optical mapping, whereas complex cases comprising simple CNVs can be confirmed by CMA, karyotyping/FISH, or digital droplet PCR.

## Concluding remarks

Clinical genomic diagnostics can identify many CGRs through the use of established methods such as classic aCGH and karyotyping/FISH. These techniques have resulted in the detection of numerous complex rearrangements over the past 30 years and have increased our knowledge of normal and disease-causing genomic variation with limited resolution. Recently, however, new methods such as short-read GS have emerged as a new tool in deciphering genomic variation. Although short-read GS technology was an improvement compared to aCGH alone, long-read GS and optical mapping are effective complementary technologies to obtain information such as the phasing of multiple *in cis* events. With increasingly higher complexity of genomic rearrangements in constitutional diseases as well as in cancer, the challenge is to use the appropriate tool to assemble a specific CGR type. By applying orthogonal approaches, the likelihood of uncovering the genomic structure increases. There is no simple solution to all cases, and the choice of methods must instead be tailored to the molecular features of the rearrangement (see Outstanding questions).

Although GS costs continue to drop, enabling studies of large cohorts, short-read and long-read GS together with optical mapping should be in the toolbox for future clinical and research laboratories – and these may be particularly important for individuals with unsolved Mendelian disorders. The more details, that are gleaned by resolving complex aberrations, the more a patient’s care can be streamlined in the pursuit of personalized medicine.

## Figures and Tables

**Table 1.** Genomic findings in large cohorts

Cohort	Cases	Unique SVs	SVs per genome	CGRs (total)	Methods	Refs
Nagasaki, M. *et al.* (2015)	1070	56 697	2699	N/a^a^	Short-read GS, CMA	[82]
Sudmant *et al.* (2015)	2504	68 818	4405	1651^b^	Long/short-read GS	[29]
Collins *et al.* (2017)	689	11 735	636	289	Long/short-read GS, CMA	[24]
Chiang *et al.* (2017)	147	23 602	3552	N/a	Short-read GS, RNA-seq	[81]
Levy-Sakin *et al.* (2019)	154	15 601	1539	934	Short-read GS, optical mapping	[30]
Abel *et al.* (2020)	17 795^c^	118 973 (GRCh37) 241 031 (GRCh38)	4442^c^	33^d^	Long/short-read GS	[25]
Collins *et al.* (2020)	14 237	335 470	7439	5295	Long/short-read GS	[23]
Ebert *et al.* (2021)^e^	31	32 627 107 136	9320 24 596	667 N/a	Long/short-read GS, optical mapping, Strand-Seq	[22]

a N/a, not available.

b Only referring to complex deletions.

c Refers to the aggregated databases from GRCh37 and GRCh38.

d Only ultra-rare SVs.

e Refers to the Illumina integration callset: top row short-read GS, bottom row long-read GS.

## Box 1. Rare diseases

In the USA a rare disease is defined as a condition that affects fewer than 200 000 people (~86/100 000) [69], whereas in the EU a rare disease is a condition occurring in one of 2000 individuals (~50/100 000) (EURORDIS, the European organization for rare disease). The combined prevalence of such rare conditions has been estimated to be between 3.5% and 5.9%, meaning that ~300 million people worldwide are living with a rare disease [70]. This heterogeneous group of diagnoses includes >8000 distinct conditions of which the majority (72%) are genetic ([70–72]; OrphaNet, http://www.orpha.net). Rare diseases can be life-threatening and chronic, many of which (~70%) manifest during early childhood, resulting in a severely diminished quality of life [70]. Difficulty in obtaining a molecular diagnosis is a common factor limiting access to proper care, management, and prevention for individuals and families with rare and undiagnosed diseases. Although these diseases may individually be classified as rare, the total number of individuals affected can be high, making the diagnosis of a rare disease a collectively common event. Therefore, increased understanding of the underlying genetic etiology of rare diseases is clinically important and key to the further development of potential treatments.

Investigating SNVs with modern sequencing technologies has been a remarkable success and has led to a diagnosis for many individuals with rare disorders [73]. In addition ~240–250 new genes per year have been associated with a disease (reviewed by Seaby *et al*. [74]). A recent review predicts that ~6000 Mendelian diseases remain to be molecularly resolved [75], and the majority of such patients lack a molecular diagnosis (i.e., unsolved families with Mendelian conditions). While SVs present a challenge to detect and interpret, they contribute to novel gene discovery and may help to end the ‘diagnostic odyssey’ in families with rare diseases [74].

## Box 2. Background genomic variation in the general population

SVs are common in healthy individuals, as exemplified by CNVs. The background structural variation in the human genome was first characterized for CNVs in studies using CMA – either SNP arrays or aCGH – and was compiled in the Database of Genomic Variants (DGV) that started in 2004 and has grown to >8 million genomic variation entries covering 86% of the genome [76,77]. More recently, a large catalogue of SVs detected by GS based on >15 700 genomes was released through the gnomAD initiative [23] and several population-specific catalogues such as SweGen (*N* = 1000) [78], the Genome of The Netherlands (*N* = 250) [19], and the Genome Denmark project (*N* = 150) [79]. In addition, several large cohorts – many including >1000 individuals – have employed short-read and long-read GS to study common variation in human genomes [80] (see Table 1 in main text).

The rapid improvement of detection algorithms involving SVs is reflected by the high number of novel SVs that continue to be detected, such as in Sudmant *et al*. where 60% of detected SVs were novel compared to the DGV [77] and 71% compared to previous releases from the 1000 Genomes Project [29]. Furthermore, cohort studies with continental group-wise comparisons reported the largest genetic diversity in the African group – which harbored 29% more heterozygous deletions than other groups [20,23,81]. A difference between populations was also observed for unmappable reads between the European and African populations [21]. By contrast, all five superpopulations – Africans, Americans, East Asians, Europeans, and South Asians – share 30–44% of large indels [30].

## Box 3. CGRs and the human reference genomes

CGRs, similar to other genomic variants, are identified based on differences between the patient and reference genome used in the analysis. The possibility to identify and resolve a specific variant depends on whether the reference genome used resembles the ancestral genome in which the CGR first occurred. The first human reference genome was completed in 2003 as part of the Human Genome Project and was named NCBI34 or hg16. Many gaps were still present that have been filled in over time. Several iterations and updates to this reference were applied, and in 2009 GRCh37/hg19 was released – which is still widely utilized today [83]. A later version is also available (GRCh38/hg38) that contains multiple haplotypes, fewer gaps, and a predicted but not yet solved centromeric sequence [84]. There are several examples of individual CGR cases where GS data, unmappable to GRCh37/hg19, were successfully aligned to the updated reference [8], proving that reference genomes are dynamic and increasingly accurate as the technology advances. Because many SVs and especially CGRs tend to involve low-complexity regions and/or segmental duplications (SDs), it is important to use a reference genome that has a good coverage in such regions. The recently released telomere-to-telomere (T2T) dataset attempts to fill gaps, bridge centromeric and telomeric regions, and correctly map large repeats [85]. However, although the T2T reference is a huge enterprise with potential impact in resolving specific disease-causing CGRs [86], it is still a haploid genome based on a single individual [86]. To fully capture and phase disease-causing SVs and SNVs, a diploid reference genome is needed. As more individuals are sequenced worldwide, variability between populations has emerged both for SNVs and SVs [23]. Regarding the latter, inter-population differences are high; although closely related groups share many of their SVs, a high fraction of SVs (86%) are exclusively observed within a single continental group [67]. As a result, population-specific databases have been generated to solve SVs. Examples of countries with such high-quality reference genomes are Sweden [87], Denmark [79], The Netherlands [88], China [89], Korea [90], and Japan [91].

## Highlights

Structural variants (SVs), particularly complex genomic rearrangements (CGRs), are underappreciated disease-causing variants.

CGRs are a group of SVs whose detection, resolution, and clinical interpretation remains challenging.

The increased use of next-generation sequencing in clinical and research laboratories is uncovering the growing relevance of CGRs to rare genomic events and their contribution to disease.

Combining standard SV detection methodologies with next-generation sequencing and genome mapping allows investigation of genomic regions prone to instability.

Studying individuals with constitutional diseases as well as with specific cancer types reveal the origins of locus-specific patterns of CGRs potentially impacting patient treatment.

## Outstanding questions

What is the occurrence rate of CGRs?

What is the contribution of CGRs to unsolved Mendelian diseases?

Do CGRs in noncoding regions contribute to gene expression variability and disease?

How can the detection of CGRs be refined to improve clinical assessment?

What are the molecular mechanisms underlying CGRs? As more clinical cases are made public and new diseases are found by employing emerging technologies, is it possible to fully understand how complex rearrangements are formed?

Is it possible to devise artificial models for complex rearrangements? The mechanisms underlying most diagnosed complex rearrangements are inferred from observation of their structural fingerprints, but validation through *in silico* simulation will be necessary to confirm the mechanism.

## Glossary

Array comparative genomic hybridization (aCGH): a molecular cytogenetic method to detect relative copy number changes (deletions and amplifications) in the genome. Depending on the design, either the entire genome or specific targeted regions can be assessed.
Balanced chromosomal rearrangements (BCRs): genomic rearrangements which only contain copy number neutral SVs such as reciprocal translocations or inversions.
Chromoanasynthesis: structural variation generated by multiple microhomology-mediated template switching during repair of single-ended double-stranded breaks.
Chromoplexy: genome shattering similar to chromothripsis affecting multiple chromosomes. It leads to multiple translocations in combination with chained rearrangements generated by a sequential-dependent mechanism.
Chromosomal microarray (CMA): a chip-based detection method to quantify relative changes in the genome. The procedure combines SNP and aCGH arrays to detect SNVs and CNVs, respectively.
Chromothripsis: a one-time catastrophic event affecting one chromosome leading to DNA shattering and subsequent short-segmented stitching in a clustered genomic region.
Complex chromosomal rearrangement (CCR): large-scale genomic rearrangement involving the exchange of material between two or more chromosomes that generates at least three cytogenetically visible breakpoints.
Complex genomic rearrangement (CGR): large-scale genomic alteration involving more than one seemingly simple SV and more than two breakpoints.
Copy number variants (CNVs): SVs with a distinct number of alleles that depart from a diploid human genome. Examples are deletions and duplications.
Coverage: measurement characteristics for genome detection methods such as genome or exome sequencing. It contains the vertical coverage (sequencing depth) which stands for the average number of reads covering the same nucleotide, and the horizontal coverage (reference coverage) representing the percentage of the reference genome covered by reads.
Genome sequencing (GS): the reformed term for massively parallel sequencing (MPS) and whole-genome sequencing (WGS); refers to the determination of genomic sequences at nucleotide resolution. Short or long DNA reads are analyzed base by base and later mapped to a reference genome for sequence alignment.
Genomic disorders: a group of genetic diseases caused by SVs mediated or stimulated by DNA segments that are prone to genomic instability.
Hybrid assembly: data from short- and long-read GS including different library preparations that can be merged into a single collection of reads to assemble a genome. The alignment to the reference genome benefits from a high horizontal and a high vertical coverage and provides an increased ability to map within repetitive sequence.
Low-copy repeats (LCRs): paralogous sequences sharing at least 97% sequence identity and >10 kb in length that can act as substrates for ectopic recombination.
Microhomology-mediated breakinduced replication (MMBIR): RAD51-independent break-induced replication mechanism that utilizes microhomology to resume replication.
Nonallelic homologous recombination (NAHR): a mechanism of nonallelic pairing and recombination of paralogous sequences that can generate genomic rearrangements.
Non-homologous end joining (NHEJ): a repair mechanism that processes and reconnects double-stranded breaks in DNA with or without microhomology. NHEJ can be accompanied by small indels at the junction.
Runs of homozygosity (ROHs): contiguous allelic segments of DNA with identical haplotypes that potentially reflect a homozygous state across all assayed genomic positions.
Segmental duplication (SD): paralogous sequences sharing 90% sequence identity and >1 kb in length.
Single-nucleotide variants (SNVs): DNA changes affecting a single DNA base (adenine, thymine, cytosine, guanine) at a specific genomic location.
Structural variant (SV): a structural change in the genome that represents the addition, removal, or relocation of a genomic sequence. SVs are most commonly defined as DNA stretches longer than 100 bp.
